# The gut microbiome drives inter- and intra-individual differences in metabolism of bioactive small molecules

**DOI:** 10.1038/s41598-020-76558-5

**Published:** 2020-11-11

**Authors:** Asimina Kerimi, Nicolai U. Kraut, Joana Amarante da Encarnacao, Gary Williamson

**Affiliations:** 1 Department of Nutrition, Dietetics and Food, School of Clinical Sciences at Monash Health, Faculty of Medicine, Nursing and Health Sciences, Monash University, Notting Hill BASE Facility, 264 Ferntree Gully Road, Notting Hill, VIC 3168 Australia; 2 School of Food Science and Nutrition, University of Leeds, Leeds, LS2 9JT UK

**Keywords:** Biomarkers, Gastroenterology, Risk factors

## Abstract

The origin of inter-individual variability in the action of bioactive small molecules from the diet is poorly understood and poses a substantial obstacle to harnessing their potential for attenuating disease risk. Epidemiological studies show that coffee lowers the risk of developing type 2 diabetes, independently of caffeine, but since coffee is a complex matrix, consumption gives rise to different classes of metabolites in vivo which in turn can affect multiple related pathways in disease development. We quantified key urinary coffee phenolic acid metabolites repeated three times in 36 volunteers, and observed the highest inter- and intra-individual variation for metabolites produced by the colonic microbiome. Notably, a urinary phenolic metabolite not requiring the action of the microbiota was positively correlated with fasting plasma insulin. These data highlight the role of the gut microbiota as the main driver of both intra- and inter-individual variation in metabolism of dietary bioactive small molecules.

## Introduction

Personalised nutrition and inter-individual variation are areas highlighted requiring further research when devising dietary and policy priorities to reduce the global crises of obesity and diabetes^1^. A prime example is the consumption of coffee, linked dose-dependently through several epidemiological studies with reduction in risk of type 2 diabetes^2^, a lowered risk of cardiovascular disease^3^ and of non-alcoholic fatty liver^4,5^. Although there remain numerous questions regarding the lack of understanding how coffee drinking is related to prevention of disease^6^ , mechanisms in vivo may involve effects on lipid^7^ and carbohydrate metabolism such as glucose absorption and utilisation^8,9^. Although coffee is clearly protective against risk of developing type 2 diabetes^2^, short term consumption puzzlingly leads to impaired glucose metabolism^10,11^. Long-term intervention studies on coffee are difficult to conduct as coffee drinkers are unwilling to give up consumption, and non-coffee drinkers are unwilling to consume coffee for the required study duration. Yet another key obstacle in harnessing scientific evidence in the roadmap to personalised nutrition are the gaps in knowledge surrounding individual responses. Inter-individual variation is becoming increasingly recognised as a confounding factor in studies on the effects of biologically active molecules including nutrients, drugs and phytochemicals^12,13^. Both interventions^14^ as well as epidemiological studies^15^ are now beginning to incorporate individual characteristics when reporting activities based on individual participant data (IPD). Less attention has been paid to the effects of temporal intra-individual variation in responses, although it has an equal potential to confound collected intervention data^16^. An important potential factor contributing to variation between individuals is the gut microbiota. The gut microbiota has been shown to be a critical mediator relating nutrition to disease risk^17^, and changes in gut microbiota have identified individuals at risk of developing type 2 diabetes^18^.

**Figure 1:**
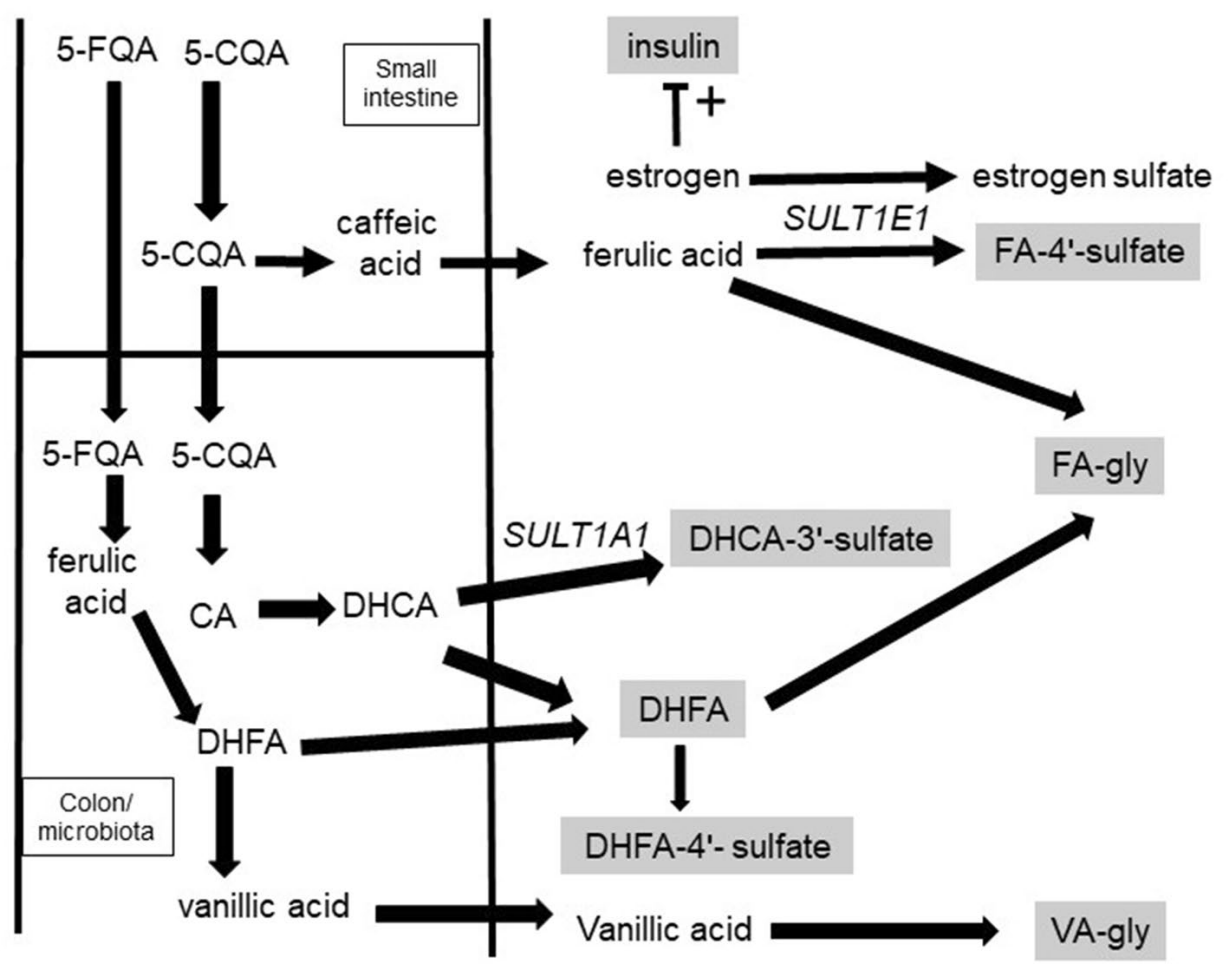
Sites of absorption and metabolism of ingested phenolic acids. Compounds indicated in boxes were measured in this study. 5-CQA is hydrolysed by pancreatic esterase(s) fivefold faster than 3 or 4-CQA, whereas 3-CQA is hydrolysed by brush border esterase(s) tenfold more rapidly than 5-CQA. FQAs are not substrates for pancreatic enzymes^56^. Both human pancreatic and brush border enzymes have relatively low activities compared to the gut microbiota esterases, which act efficiently on all chlorogenic acids^60^. CGAs are partially hydrolysed in the small intestine^56^, and lead to metabolites such as FA-4′-sulfate in the blood after 1–2 h^27,61^. The majority of CGAs pass along the small intestine unmodified and, following microbial hydrolysis in the colon, are converted to metabolites such as 3-(4′-hydroxy-3′-methoxyphenyl)propanoic acid (DHFA), 3-(3′,4′-dihydroxyphenyl)propanoic acid-3′-sulfate (DHCA-3′-sulfate) and 3-(3′-methoxyphenyl)propanoic acid-4′-sulfate (DHFA-4**′-**sulfate) which appear in the blood after > 4 h^27^. These lower molecular weight compounds are ultimately excreted in the urine, together with glycine conjugates^27^.

**Figure 2:**
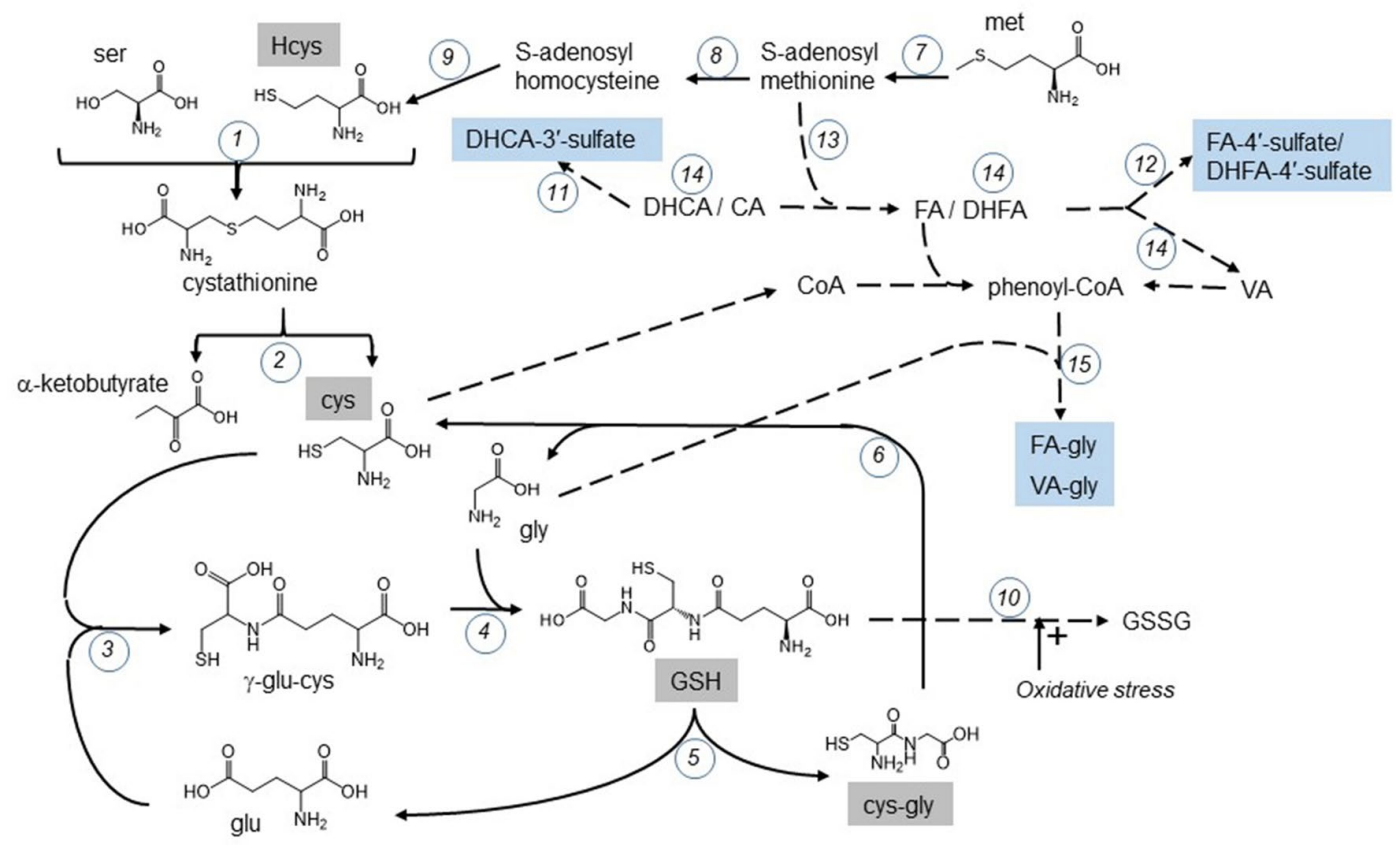
Relationship and overlap between metabolism of phenolic acids and aminothiols. Enzymes shown by circled numbers: 1. vitamin B6-dependent cystathionine β-synthase; 2. cystathionine γ –lyase; 3. γ-glutamylcysteine synthetase; 4. GSH synthetase; 5. γ-glutamyltransferase; 6. dipeptidase; 7. Met S-adenosyltransferase; 8. glycine N-methyltransferase; 9. S-adenosylhomocysteine hydrolase; 10. GSH peroxidase; 11. sulfotransferase SULT1A1; 12. sulfotransferase SULT1E1; 13. Catechol-*O*-methyl transferase, COMT; 14. enzymes from gut microbiota; 15. glycine N-acetyltransferase, GLYAT. Solid arrows show direct conversion, dash arrows show multi-step conversions. Met = methionine; Glu = glutamate; GSH = L-glutathione; GSSG = oxidized GSH. Compounds with names in boxes were measured in this study.

A cup of coffee is a mixture of thousands of compounds which, following consumption, undergo chemical modification in the small intestine and by the microbiota in the colon, giving rise to a multitude of metabolites quantitatively varying between individuals. Many of the effects of coffee are irrespective of the caffeine content and therefore attributable to constituent chlorogenic acids^19,20^ (CGAs; supplementary Figure 1). For example, in mildly hypertensive patients, CGAs as pure compounds reduced blood pressure^21,22^, lowered plasma HCys^23^ and improved endothelial function^24,25^. The metabolism of CGAs after coffee consumption has been well defined and characterized^19,26^, and sites of metabolism and absorption are clear^27,28^ (Fig. 1). Phenolic acid and thiol metabolism share common substrates, including supply of glycine for conjugation, S-adenosyl methionine for methylation, CoA synthesis and utilization, and consumption of GSH in oxidative stress, pointing to an extended, largely unexplored network of potential interactions between metabolism of coffee phenolic acid constituents and circulating thiols (Fig. 2).

In this paper, we exploit the available detailed knowledge of the pathways and mechanisms of absorption of CGAs to provide a novel and unique avenue to studying the role of the gut microbiota in inter- and intra-individual variation, and to help understand how the metabolism might be related to blood thiol and other related biomarkers.

## Results

### Characterization of the volunteers

**Figure 3:**
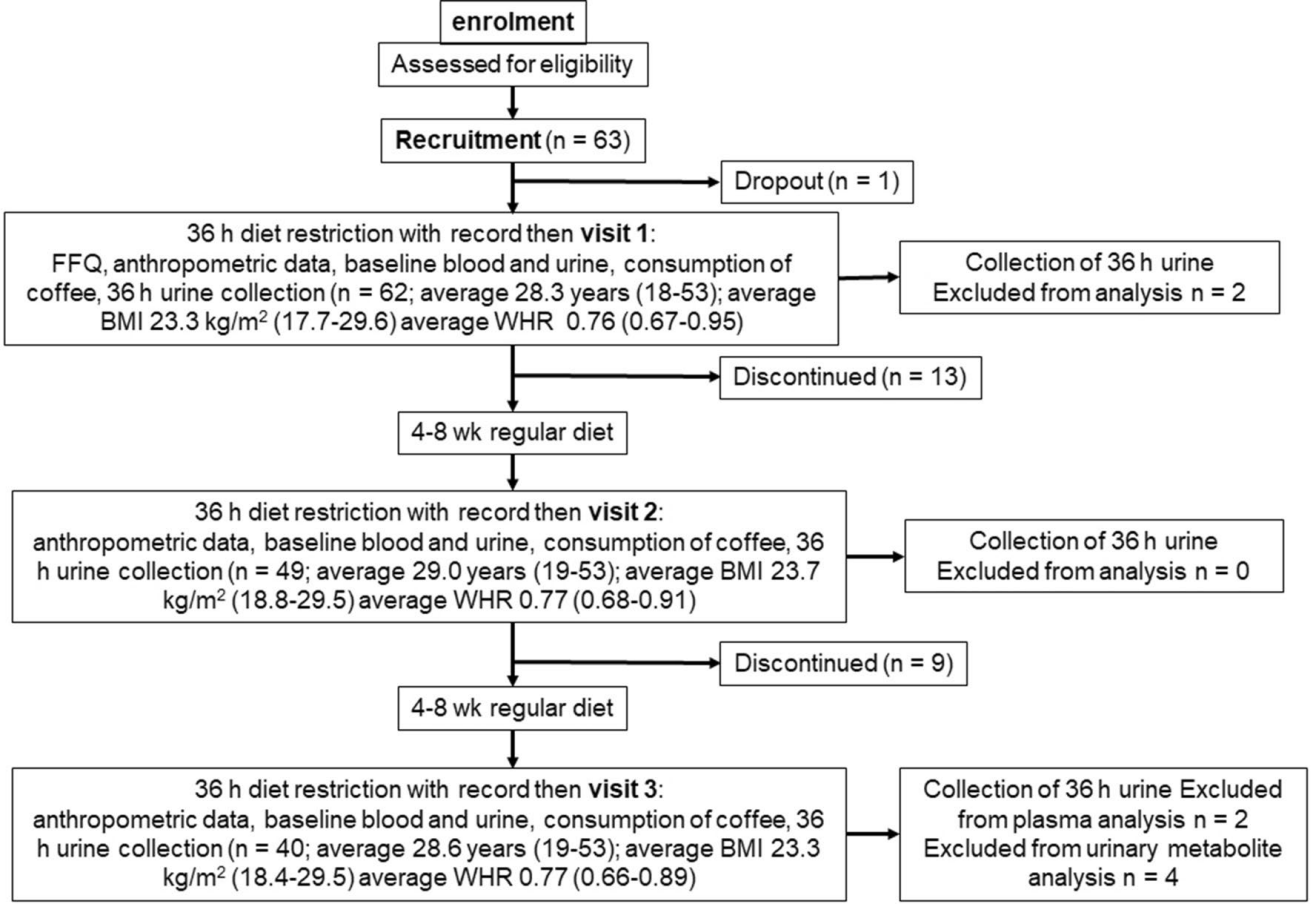
Design of the intervention study. HAQ, health assessment questionnaire; FFQ, food frequency questionnaire.

The study design is summarised in Fig. 3. Anthropometric and blood biomarker measurements in volunteers (final n = 38) remained stable and unchanged between the three visits (Table 1). Notable differences in the concentrations of biomarkers between males and females are shown in Supplementary Figure 2. Cys and CysGly were 9% (*p* = 0.003) and 13% (*p* = 0.05) higher respectively in males, in agreement with previous data^29^. HCys was 30% higher (*p* = 0.002), and, as expected^30^, uric acid was 35% higher in males (*p* < 0.0005).

**Table 1:** Baseline characteristics of the volunteers at each of the visits.

	Visit 1	Visit 2	Visit 3
Weight (kg)	65.9 ± 10.1	66.1 ± 9.7	66.3 ± 9.8
Height (m)	1.69 ± 0.09	1.69 ± 0.09	1.69 ± 0.09
BMI (kg/m^2^)	23.13 ± 2.96	23.19 ± 2.90	23.28 ± 3.08
Waist (cm)	75.2 ± 8.4	75.5 ± 7.3	75.2 ± 7.7
Hip (cm)	98.1 ± 6.9	98.2 ± 6.6	98.1 ± 6.8
Waist to hip ratio	0.77 ± 0.07	0.77 ± 0.06	0.77 ± 0.06
Systolic BP (mmHg)	116 ± 8	114 ± 7	115 ± 9
Diastolic BP (mmHg)	71 ± 6	72 ± 6	71 ± 6
tCys (μmol/L)	260 ± 27	261 ± 25	260 ± 26
tHCys (μmol/L)	9.9 ± 4.7	9.3 ± 3.9	9.5 ± 4.0
tCysGly (μmol/L)	32.8 ± 6.4	29.5 ± 5.3	30.4 ± 6.6
tGSH (μmol/L)	8.9 ± 2.6	7.4 ± 1.4	7.4 ± 1.8
Glucose (mmol/L)	4.7 ± 0.3	4.8 ± 0.4	4.8 ± 0.4
Insulin (pmol/L)	52.1 ± 14.1	55.0 ± 16.4	58.0 ± 18.4
Uric acid (mmol/L)	0.26 ± 0.06	0.26 ± 0.05	0.26 ± 0.08

Characterization of the participants that completed the full three visits (n = 38), with data presented for each visit as the mean ± standard deviation (SD). Values were not significantly different between visits (*p* > 0.05; parametric ANOVA with repeated measures). Data in fasting plasma are comparable to published values (μmol/L): Cys: 219 ± 14^62^; 256 ± 17^63^; 321 ± 50^64^; for HCys: 9.5 ± 0.4^62^; 9.9 ± 2.4^65^ ; 14.1 ± 4.9^64^; for CysGly: 24.4 ± 4.5^64^; 39.7 ± 4.9^62^; for GSH: 2–20^66^; 5.69 ± 1.33^64^; 9.07 ± 1.55^62^. t = total. BP, blood pressure.

### Analysis of coffee chlorogenic acids

As determined by LC-MS, the content of caffeoyl-quinic acids (sum of 3-CQA, 115.9 µmol, 4-CQA, 195.9 µmol and 5-CQA, 291.9 µmol), feruloyl-quinic acids (sum of 3-FQA, 18.9 µmol, 4-FQA, 38.9 µmol and 5-FQA, 110.7 µmol) and of dicaffeoyl-quinic acids (sum of 3,4-diCQA, 67.5 µmol and 3,5-diCQA, 42.1 µmol) in 4 g of the administered instant coffee was ~ 215, 63 and 51 mg respectively (330 mg total chlorogenic acids; 882 ± 37 µmol).

### Inter-individual variation of phenolic acid conjugate profiles in urine

The six major urinary metabolites of caffeoylquinic acids (CQAs), feruloylquinic acids (FQAs) and derivatives, namely 3-(3′,4′-dihydroxyphenyl)propanoic acid-3′-sulfate (DHCA-3′-sulfate), feruloylglycine (FA-gly), vanilloylglycine (VA-gly), 3′-methoxycinnamic acid-4′-sulfate (FA-4′-sulfate), 3-(4′-hydroxy-3′-methoxyphenyl)propanoic acid-4′-sulfate (DHFA-4′-sulfate) and 3-(4′-hydroxy-3′-methoxyphenyl)propanoic acid (DHFA) (see supplementary Figure 1), were analyzed in urine at time zero (baseline) and in time periods of 0–4, 4–8, 8–12, 12–24 and 24–36 h after coffee consumption (supplementary Figure 3). Only low amounts of metabolites of caffeoylquinic acid and its derivatives were detected in baseline urine compared to the amounts excreted post-coffee consumption (Table 2). The magnitude of the standard deviation illustrates the substantial inter-individual variation observed where individual metabolites span a range between 7.5- and 36.3-fold. The amounts of metabolites in urine as a percentage of dose are comparable to previous studies on coffee, although VA-gly was not reported before (Supplementary Table 1).

**Table 2:** Inter-individual variation in urinary total (36 h) phenolic acid metabolites after coffee consumption (n = 36).

	Baseline	Range after coffee consumption			Average	CV
	(µmol)	Lowest (µmol)	Highest (µmol)	Difference (-fold)	Mean ± SD (µmol)	(%)
FA-4′-sulfate	0.9 ± 2.5	9.9	74.3	7.5	35.6 ± 19.2	72.6
DHFA-4′-sulfate	0.4 ± 1.0	4.4	67.5	15.3	24.7 ± 17.9	72.1
DHCA-3′-sulfate	0.8 ± 1.6	7.1	257.7	36.3	68.3 ± 54.8	80.3
DHFA	0.2 ± 0.4	3.3	46.8	14.2	17.1 ± 11.3	66.0
FA-gly	1.2 ± 2.2	11.3	110.3	9.8	50.6 ± 22.9	45.2
VA-gly	0.8 ± 2.4	7.4	123.3	16.6	42.0 ± 30.1	71.7
Total		68.5	435	6.4	238.2 ± 109.5	45.9

### Intra-individual variation in urinary phenolic acid conjugates

**Figure 4:**
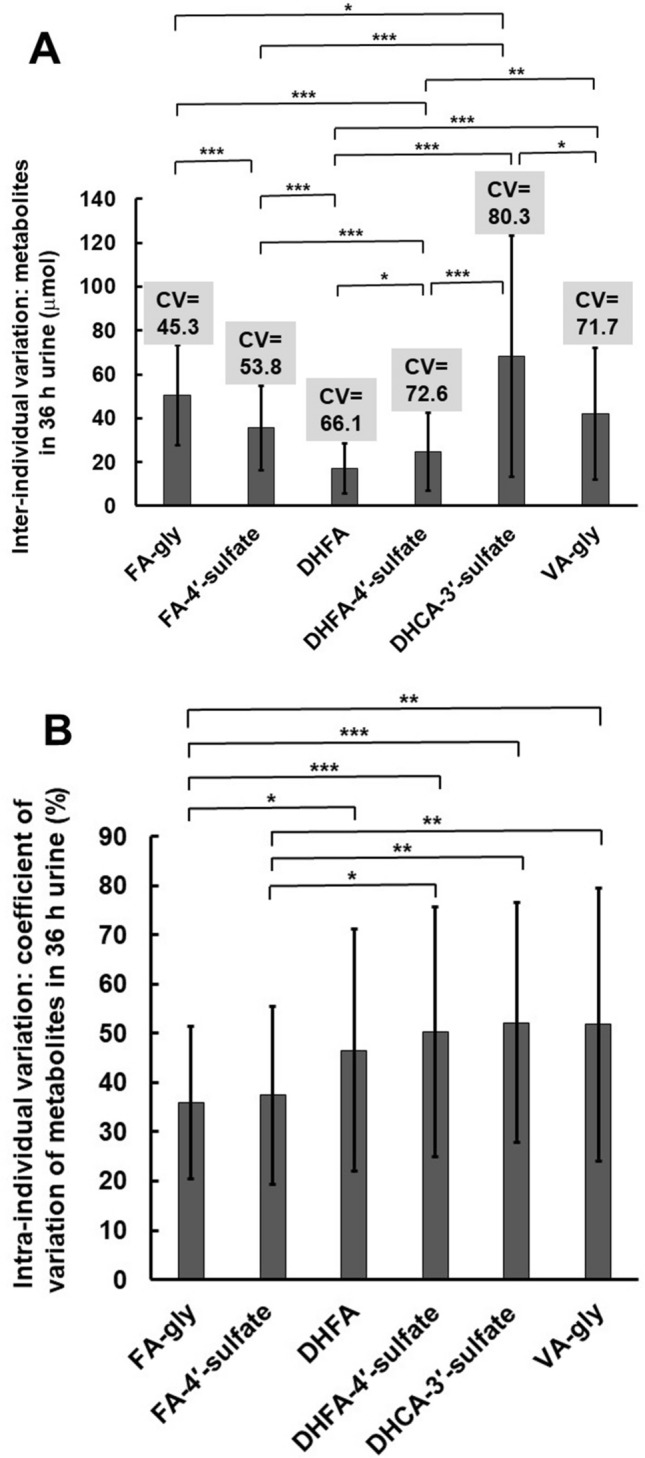
Inter-individual and temporal intra-individual variation in excretion of metabolites from coffee. (**A**) Excretion of six major urinary metabolites over 36 h after coffee consumption (n = 38) showing inter-individual variation. Values in grey boxes above columns indicate coefficient of variation (CV, %). (**B**) Excretion of six major urinary metabolites over 36 h after coffee consumption (n = 38) showing the CV of intra-individual metabolite concentrations, with error bars indicating standard deviation of CV values.* < 0.05; ** < 0.01; *** < 0.001, from student paired t-test.

The total amount of each metabolite and the sum of all measured phenolic acid metabolites in 0–36 h urine was calculated for each of the three visits and plotted per volunteer for all the fully compliant 36 volunteers who completed the study (supplementary Figure 4). Using these data, the intra-individual variation for the three visits was calculated for each volunteer, and used to estimate the mean CV and standard deviation for each metabolite (Fig. 4). The metabolites on the left (FA-gly and FA-4′-sulfate) are predominantly from endogenous metabolism, involving partial hydrolysis of chlorogenic acids in the luman of the gut to free phenolic acids, followed by absorption in the small intestine. The resulting phenolic acids are either methylated, conjugates with sulfate or with glycine. The other products, DHFA, DHCA and VA, are all derived mostly from gut microbiota metabolism, followed by absorption and conjugation by endogenous enzymes.

### Correlation between different phenolic acid conjugates in urine

Comparing the mean amount of each metabolite in each volunteer, it is apparent that all measured sulfated metabolites have a weak to moderate correlation with each other over all volunteers, indicating that individuals with high ability to sulfate will be able to do this on all phenolics (Table 3).

**Table 3:** Correlation between amounts of metabolites in urine based on IPD.

	DHCA-3′-sulfate	FA-gly	VA-gly	DHFA	DHFA-4′-sulfate
FA-gly	0.468				
VA-gly	0.018	0.032			
DHFA	0.132	0.025	0.042		
DHFA-4′-sulfate	0.474	0.152	0.015	0.161	
FA-4′-sulfate	0.241	0.266	0.000	0.028	0.292

Correlation coefficients are r^2^ values derived from simple linear regression for 36 h urine averaged over the three visits (n = 36). Weak is defined from 0.2 to 0.4, and moderate above 0.4.

### Correlation between habitual coffee consumption and biomarkers

Table 4a shows correlations between habitual coffee and fruit consumption with excretion of urinary metabolites. Tea and vegetable consumption, which are much lower in phenolic acids compared to coffee and fruits, showed no correlation with any of the metabolites. DHCA-3′-sulfate and DHFA-4ʹ-sulfate in urine decreased with higher coffee consumption, whether or not “coffee level” (frequency of coffee consumption combined with an estimate of coffee strength) or number of cups of coffee per week were considered. Fruit, which contains phenolic acids but at a lower overall dietary level than coffee, correlated with lower FA-gly in urine. Coffee consumption as indicated by “coffee level” was associated with increased plasma Cys, HCys, CysGly and GSH concentrations (Table 4b).

**Table 4:** Correlation between habitual coffee consumption and (a) urinary phenolic acid metabolites after acute coffee intake or (b) fasting blood parameters.

(a)	sum of all metabolites	DHCA-3′-sulfate	FA-gly	DHFA-4′-sulfate					
Cups of coffee per week									
r_s_	*− 0.338	**− 0.468	ns	*− 0.354					
*p*	0.038	0.003	ns	0.029					
“Coffee level”									
r_s_	*− 0.332	**− 0.464	ns	*− 0.390					
*p*	0.042	0.003	ns	0.016					
Portions of fruit per week									
r_s_	*− 0.370	ns	r_p_ = *− 0.385	ns					
*p*	0.022	ns	0.017	ns					

(b)	Cys	HCys	CysGly	Cys:CysGly	GSH	GSH:CysGly	Uric acid	Glucose	Insulin
“Coffee level”									
r_s_	*0.326	*0.300	*0.312	ns	*0.307	ns	ns	ns	ns
*p*	0.018	0.030	0.025	ns	0.027	ns	ns	ns	ns

r_s_: Spearman’s coefficient, unless labeled by r_p_: Pearson’s coefficient; significance levels, *p* ≤ 0.05*, *p* ≤ 0.01** (n = 36). gly = glycine. ns = no significant relationship. VA-gly, FA-4′-sulfate and DHFA showed no correlation with habitual consumption categories, and so are not shown. No significant correlations were found between plasma biomarkers and cups of coffee or tea per week, nor fruit and vegetable consumption.

### Correlations of anthropometric data with plasma aminothiol biomarkers

Uric acid, Cys and HCys concentrations increased with both higher BMI and higher waist to hip ratio, and glucose and insulin also increased with higher BMI (supplementary Table 2) in agreement with various studies on biomarkers unrelated to coffee consumption^31,32^ HCys and CysGly, as well as the GSH/CysGly and CysGly/Cys ratios, increased with increasing systolic blood pressure (supplementary Table 2). Volunteers with higher insulin also had higher glucose (Pearson correlation (Pc) = 0.308; *p* = 0.014); GSH was correlated with insulin (Pc = 0.402; *p* = 0.014) and with CysGly (Pc = 0.283; *p* = 0.025); uric acid was correlated with Cys (Pc = 0.363; *p* = 0.003), HCys (Pc = 0.422; *p* = 0.001) and with CysGly (Pc = 0.344; *p* = 0.006); and Cys was correlated with HCys (Pc = 0.463; *p* < 0.001).

### Correlations of urinary phenolic acids with plasma aminothiol biomarkers

To assess pathway relationships shown in Fig. 2, we explored whether there were any correlations between the urinary metabolites and the blood biomarkers (Table 5). The most robust outcome was that urinary FA-4′-sulfate, the formation of which does not require gut microbiota, was strongly correlated with fasting insulin (*p* = 0.009). Both microbe-derived sulfate metabolites, DHCA-3′-sulfate and DHFA-4′-sulfate, were correlated with plasma CysGly concentration while VA-gly excretion was correlated with plasma HCys concentration (*p* = 0.018).

**Table 5:** Correlations between phenolic acid metabolite excretion and plasma biomarkers.

	DHCA-3′-sulfate	VA-gly	FA-4′-sulfate	DHFA-4′-sulfate
HCys				
r_s_	ns	*0.388	ns	ns
*p*		0.018		
CysGly				
r_s_	*− 0.340	ns	ns	*− 0.345
*p*	0.037			0.034
Insulin				
r_s_	*0.328		**0.417	
*p*	0.044	ns	0.009	ns

r_s_: Spearman’s coefficient; *p* ≤ 0.05*, *p* ≤ 0.01**. N = 36. Sum of all metabolites, FA-gly and DHFA showed no correlations with plasma biomarkers. Cys, GSH, uric acid and glucose showed no correlations with phenolic metabolites.

## Discussion

The concept of what constitutes a healthy diet is mostly assessed from the point of view of lowering the risk of developing diseases such as obesity and type 2 diabetes. Nonetheless, what regime may work for certain individuals may not prove effective for others due to inter-individual variation. Here we show that both intra- and inter-individual variation in coffee chlorogenic acid metabolism was greatest when metabolite formation required the participation of the colonic microbiota. In caged laboratory mice, inflammation, genotype and inter-individual effects drove gut microbiota variation^33^ and the gut microbiota has a strong influence on consistency and perceived success of a dietary intervention^34^. Conversely it has been proposed that the gut microbiota might be a more accessible way of modulating health compared to host-derived factors^1^.

An example of a metabolite where formation does not require the involvement of colonic microbiota is FA-4′-sulfate. This early-appearing peak is due to sulfation of ferulic acid; the latter is a product of small intestinal hydrolysis (non-microbial) of chlorogenic acids followed by conjugation by catechol-*O*-methyltransferase (COMT) and sulfotransferase (SULT)1E1, as endogenous human enzymes. In comparison, formation of DHFA, DHCA and VA require microbial catabolism of the original chlorogenic acid, and all of these compounds and conjugates exhibit greater inter- and intra-individual variation than the ferulic acid conjugates. Several factors contribute to inter-individual variation in bioavailability including gut microbiota composition, but also host-specific factors such as polymorphisms in metabolic enzymes and transporters (such as SULTs, UGTs, OAT and ABC transporters), variation in glycine conjugation, and adaptation of metabolic pathways to the habitual diet^13,35^. SULT1A1 is active on caffeic acid and DHCA, whereas SULT1E1 is more active on FA and DHFA^36^, and both of these enzymes have known polymorphisms^37,38^ which affect activity leading to host-derived inter-individual variation. All measured sulfated metabolites showed a weak to moderate correlation with each other, indicating that individuals with high ability to sulfate are able to similarly process all phenolics through this route. The natural substrate of SULT1E1, estrogen, is also a regulator of insulin action and mitochondrial function. Estrogen receptor-α elicits the metabolic effects of estrogen by genomic, non-genomic, and mitochondrial mechanisms that regulate insulin signalling, substrate oxidation, and energetics^39^ while sulfation leads to its inactivation^40^. The fact that urinary FA-4′-sulfate strongly correlated with higher fasting insulin here may suggest an interplay between sulfation and insulin levels mediated through modulation of SULTs. In support of this, SULT1E1 activity was enhanced in the presence of insulin-like growth factor IGF-1 in HUVECs^41^, and insulin increased the related enzyme SULT1A1 in HT29 cells^42^. In addition, hepatic insulin sensitivity was improved in SULT1E1-over-expressing transgenic mice, while muscle insulin sensitivity did not change^43^. These data point to an underlying relationship between phenolic acid sulfation and estrogen activity and inactivation. The CGA urinary metabolites, DHCA-3′-sulfate and DHFA-4′-sulfate, decreased with higher coffee consumption, indicating adaptation. Both products require the action of gut microbiota, and so the colonic bacteria could adapt to increasing levels of substrate by induction of the relevant metabolising enzymes.

Aminothiols play a critical role as regulators of the organism’s redox status, and are metabolically inter-related via the trans-sulfuration pathway, linking methionine to the biosynthesis of glutathione. Increases in plasma HCys are an independent risk factor for premature cardiovascular conditions^44^, and dysbalance in the trans-sulfuration pathway could be partly explained by reduced enzymatic activities that accompany aging, as well as an impaired renal function^45^. CysGly has been positively correlated to oxidized LDL, myocardial infarction, total HCys, total Cys, diastolic blood pressure and BMI^46^, and the HCys:Cys and GSH:CysGly ratios were positively and negatively associated with the cardiovascular risk score, respectively^47^. Blood glucose and insulin are markers of sugar metabolism and insulin resistance. Markers which are highly controlled through endogenous metabolism, such as Cys, HCys, CysGly, glutathione, glucose and insulin, showed almost no temporal intra-individual variation over the 3 visits in the absence of a specific intervention (Table 1). Here we show that coffee consumption was associated with increased plasma Cys, HCys, CysGly and GSH concentrations. Previously, plasma HCys increased in volunteers who drank coffee for 4 wk^48^, and was also higher in coffee consumers, exacerbated by smoking, in The Hordaland Homocysteine study^49^. Habitual coffee consumption did not affect baseline CysGly^50^, but the consumption of coffee increased the total plasma GSH^51^. A review of intervention studies on coffee reached the same conclusion^52^.

Coffee is a complex beverage which is consumed extensively worldwide. Dissecting out its effects on health is not easy and requires multiple approaches. The novel approach we have taken here has linked metabolism of coffee constituents to health biomarkers, and has most importantly highlighted the large contribution that the colonic microbiota make to both intra- and inter-individual variation in the metabolism of bioactive small molecules.

## Materials and methods

### Sources of chemicals

Cystamine dihydrochloride, cysteamine (≥ 98%), Cys (≥ 98%), CysGly (≥ 85%), reduced GSH (≥ 98%), HCys (≥ 95%), acetic acid, sodium acetate, boric acid, trichloroacetic acid, EDTA and phosphate buffered saline were purchased from Sigma-Aldrich (Dorset, UK). Sodium hydroxide (50% concentrated solution) and 7-fluorobenzofurazan-4-sulfonic acid were from Fluka (Sigma-Aldrich). Analytical reagent-grade hydrochloric acid and HPLC-grade methanol were from Fisher Scientific (Loughborough, UK) and tris(2-carboxyethyl)phosphine hydrochloride was from Thermo Fisher Scientific (Waltham, MA, USA). Milli-Q water (18.2 MΩ cm^−1^) was used in all experiments (Merck Millipore UK, Watford, UK). Sulfate conjugates were synthesized as previously described^53^. FA-gly and VA-gly were chemically synthesized and fully characterised, and this will be published elsewhere.

### Analytical methods

Glucose and insulin were measured as previously described^54^. Uric acid was quantified enzymatically^55^. Chlorogenic acids in coffee were analyzed by a modification of a published chromatographic method^56^. In brief, coffee samples were kept at 4 °C and 5 μL of prepared samples were injected in duplicate. The flow rate was set to 0.26 mL/min for a total run time of 65.1 min. The gradient started with 0% mobile phase B for 17.3 min, was increased to 25% up to 51.0 min, further increased to 100% from 51.1 to 56.0 min and dropped to 0% from 56.1 min for 10 min for re-equilibration. The Eclipse plus C18 column (2.1 mm × 100 mm, 1.8 μm pore size; Agilent Technologies, Germany) was thermostated at 30 °C. For the mass spectrometric analysis, the heated electrospray ionisation source was operated in negative mode, the source temperature set to 350 °C, with nebulizer flow at 10 L/min and a pressure of 60 psi. Multiple reaction monitoring (MRM) settings were optimised using commercially available standards at a concentration of 10 μg/mL. The flow of the first 4 min was diverted to waste.

### Urine collection during the intervention studies

Urine samples were collected into sterilised unisex urine collection containers containing 1 g ascorbic acid for 0, 0–4, 4–8 and 8–12 h and 3 g ascorbic acid for 12–24 and 24–36 h, respectively. Within 48 h after collection, the urine volume was determined by weight (1 ml = 1 g), an aliquot of 45 mL centrifuged at 4 °C for 10 min, and 12 mL of the supernatant retained. Sodium azide was added from an aqueous solution to a final concentration of 0.1% prior to freezing the samples at − 20 °C.

### Quantification of phenolic acid conjugates in urine

Prior to LC–MS analysis, urine samples were defrosted overnight in a fridge and aliquots of 900 μL from each sample were taken. An internal standard of 100 μM sinapic acid containing 1 mM ascorbic acid prepared in mobile phase A was added (100 μL) and samples were vortexed for 10 s, inverted twice, and centrifuged at 20,000 g at 4 °C for 10 min. The supernatant was filtered through a 0.22 μm PTFE filter into amber vials. To determine metabolite recovery, sinapic acid (2–100 µM) was added as internal standard to urine samples prepared in triplicate. Samples were injected onto an Agilent ZORBAX Eclipse Plus Rapid Resolution C18 column (100 mm × 2.1 mm i.d., 1.8 μm) with a flow rate of 0.4 mL/min and total run time 25 min. Mobile phase A consisted of 95% water, 5% acetonitrile and 0.1% formic acid, mobile phase B of 95% acetonitrile, 5% water and 0.1% formic acid. The gradient consisted of 0% mobile phase B for the first 12 min, increased to 40% at 16 min, further increased to 100% at 16.5 min and kept until 19.5 min prior to equilibration with 0% B from 20.0 to 25 min. The column was thermostated at 35 °C. For the mass spectrometric analysis, the heated electrospray ionisation source was operated in negative mode with a source temperature set to 350 °C, a nebulizer nitrogen flow of 13 L/min and a pressure of 60 psi. The eluent flow of the first 3 min was diverted to waste.

### Mass spectrometric parameters for urinary metabolites

Multiple reaction monitoring (MRM) settings were optimised for the individual compounds (Supplementary Table 3). Urinary metabolites were quantified using external calibration, from 10 nM to 50 μM in mobile phase A containing 1 mM ascorbic acid. The limit of detection (LOD) and the limit of quantitation (LOQ) were calculated using LOD = (3.3*intercept)/slope and LOQ = (10*intercept)/slope. The linearity of the calibration curves was R^2^ ≥ 0.99.

### Measurement of thiols in plasma

Fasted blood was collected from the antecubital vein by venepuncture. Tubes were placed on ice and centrifuged (10 min, 3,000 g, 4 °C) within 30 min from collection. The plasma was carefully separated, aliquoted and stored at − 80 °C until analysis. Plasma processing and aminothiol analysis followed a previously described method^57^. Plasma (50 μL), phosphate buffered saline (25 μL) and cystamine dihydrochloride (25 μL, 66 μM) were mixed by vortexing before centrifugation and addition of tris (2-carboxyethyl) phosphine hydrochloride (10 µL)^49,50^. Samples were incubated for 15 min at 37 °C in a shaking water bath, cooled to room temperature, 100 g/L trichloroacetic acid containing 1 mM EDTA (90 μL) added, vortex-mixed for 1 min and then centrifuged for 10 min (13,000 g at 4 °C). Supernatant (50 μL) was added to 7-fluorobenzofurazan-4-sulfonic acid (1 g/L; 50 μL), 0.125 M borate buffer pH 9.5 containing 4 mM EDTA (127 μL) and NaOH (1.55 M; 8 μL). Five randomly selected samples were used to check that the pH for derivatization was within range (pH 9–10). Samples were briefly vortex-mixed, centrifuged and incubated in the dark for 60 min at 60 °C in a shaking water bath at 130 rpm. Samples were subsequently allowed to cool to room temperature before centrifugation (10 min, 4 °C, 13,000 g) and filtering (0.2 µm PTFE filter) into amber vials. An Agilent 1200 series HPLC equipped with a fluorescence detector was used and samples (10 μL) were injected on a Spherisorb ODS1 C18 column (5 μm particle size, 4.6 mm × 250 mm length, Waters, UK). The separation of the fluorescent aminothiol adducts was achieved isocratically (22.5 min) employing a flow rate of 0.6 mL/min at 29 °C and 0.1 M sodium acetate/acetic acid containing 0.125% methanol (pH 4.99). Detection followed excitation at 385 nm and emission at 515 nm. Chromatographic separation of the aminothiols is shown in supplementary Figure 5.

The concentration of thiols was estimated according to standard curves prepared in plasma, although the regression lines were not significantly different from calibration curves prepared in water. Data used were from 6 separate standard curves prepared and analyzed on different days. For Cys, 2 nmol injected gave an inter-assay variation (CV) of 14.4%, and 0.001 nmol gave a CV of 17.1%. Comparable values were found for the other thiols. The limits of detection and quantification were: Cys, 2, 7; HCys, 1, 3.6; CysGly, 0.6, 2; GSH, 1, 5.8, pmol on column respectively. Recoveries from spiked plasma samples were (% ± SD): Cys, 93 ± 6; HCys, 94 ± 4; CysGly, 94 ± 4; GSH, 67 ± 13.

### Eligibility criteria and participant characteristics

We confirm that all methods were carried out in accordance with relevant guidelines and regulations as follows: The study was approved by the University of Leeds, UK ethics committee (MEEC 10–035) and registered at clinicaltrials.gov (NCT01912144). Following written informed consent, volunteers were assessed as eligible if they were in general good health, between 18 and 70 y of age, BMI 18–29 kg/m^2^, not diagnosed with any chronic disease or haemophilia, had not previously undergone a gastrointestinal tract operation, were not under any long-term prescribed medication (except contraceptive), not pregnant nor lactating, non-smokers or smoking < 5 cigarettes per day and consumed ≤ 4 units of alcoholic beverages on a daily and regular basis. Suitable volunteers completed a self-assessed food frequency questionnaire before the first visit, according to the Nutritional Epidemiology Group, University of Leeds, UK^58^. Each participant was assigned a code and all biological samples and data collected were blinded. A total of 63 participants were recruited of which 62, 49, and 38 completed phase 1, phase 2 and phase 3, respectively.

### Design of the intervention study

For each phase of the study, participants were required to undergo a 36-h washout period prior to the study visit and following attendance, a 36-h period with the same dietary restrictions until all samples were collected. A 72-h dietary record of the type and quantities of all the consumed foods and beverages, other than water, was used to certify participant compliance with the diet restrictions. On the morning of the first study visit, the participants arrived fasted overnight from 10 pm and were allowed to drink water ad libitum. Fasting blood (10 mL) was withdrawn by venepuncture of the antecubital vein. Anthropometrics and blood pressure (repeated 3 times) were recorded. A baseline urine sample was collected before the polyphenol-poor breakfast was consumed along with a cup of 4 g "Green Blend" Nescafé instant Coffee (Nestlé, Vevey, Switzerland) dissolved in water (~ 300 mL boiled and allowed to cool for > 10 s). Total urine from 0 to 4, 4 to 8, 8 to 12, 12 to 24 and 24 to 36 h after coffee consumption were collected separately into containers. At the end of the 36 h urine collection, the volume of all the urine samples was noted and aliquots were centrifuged and stored at − 20 °C until analysis. The second and third visits followed the same procedure.

### Food frequency questionnaire

The FFQ used previously^58^ was modified for this study. Beverage consumption information was reported as the number of cups consumed in 7 d. For each, 6 options of answers, ranging from "none" to "more than 8 cups per day" were offered. The use of a “coffee level” was designed to take the strength of coffee into account, not just the number of cups per day. This has recently been recommended^59^. An index of strength was attributed to each strength of coffee, namely "1" for weak, "2" for medium, and "3" for strong. To determine the total "coffee level" per week for a participant, the index was then multiplied by the number of cups of the corresponding strength consumed on a weekly basis. For the consumption of fruit and vegetables, only the most commonly used products in the UK were included. The given options of consumption for vegetables and fruits ranged from "none" to "more than 5 times daily" and the unit was a portion, which was defined as the amount of product that was the size of the first of the participant.

### Statistics

When the biomarkers were analyzed by gender (all samples), no outliers were detected upon inspection of box plots and the distribution was normal in both groups for Cys (females: *p* = 0.858, males: *p* = 0.772), CysGly (females: *p* = 0.126, males: *p* = 0.873), glucose (females: *p* = 0.769, males: *p* = 0.591) and uric acid (females: *p* = 0.284, males: *p* = 0.974). For these pairs, the association with gender was tested by a parametric independent samples t-test. The results of a Levene's test indicated that equality of variances between genders was met for Cys (*p* = 0.671), CysGly (*p* = 0.644), GSH (*p* = 0.259), glucose (*p* = 0.612), insulin (*p* = 0.339), and uric acid (*p* = 0.067). A non-parametric Mann–Whitney U-test was used to assess the difference between genders for the fasting concentrations of HCys, GSH, and insulin. For these variables, outliers were present and normality was not met. Following exclusion of data from two non-compliant volunteers, the urine metabolite data were reanalyzed and a normal distribution was found. The null hypothesis was accepted with *p* = 0.352 for the Shapiro–Wilk test. Where other statistical tests were employed, they are indicated throughout the manuscript.

## Supplementary information

Supplementary Information.

Supplementary Figure s1.

Supplementary Figure s2.

Supplementary Figure s3.

Supplementary Figure s4a.

Supplementary Figure s4b.

Supplementary Figure s4c.

Supplementary Figure s5a.

Supplementary Figure s5b.

## Abbreviations

BMI: Body mass index
CGA: Chlorogenic acid
CQA: Caffeoylquinic acid
Cys: L-cysteine
CysGly: L-cysteinylglycine
CV: Coefficient of variation
DHCA: 3-(3′,4′-Dihydroxyphenyl)propanoic acid (dihydrocaffeic acid)
DHCA-3′-sulfate: 3-(4′-Hydroxyphenyl)propanoic acid-3′-sulfate (dihydrocaffeic acid-3′-sulfate)
DHFA: 3-(4′-Hydroxy-3′-methoxyphenyl)propanoic acid (dihydroferulic acid)
DHFA-4′-sulfate: 3-(3′-Methoxyphenyl)propanoic acid-4′-sulfate
diCQA: Dicaffeoylquinic acid
EDTA: Ethylenediaminetetraacetic acid
FA: 4′-Hydroxy-3′-methoxycinnamic acid (ferulic acid)
FA-gly: Feruloylglycine
FA-4′-sulfate: 3′-Methoxycinnamic acid-4′-sulfate (ferulic acid-4′-sulfate)
FQA: Feruloylquinic acid
GSH: L-glutathione
HCys: D,L-homocysteine
IPD: Individual participant data
VA: 4-Hydroxy-3-methoxybenzoic acid (vanillic acid)
VA-gly: Vanilloylglycine
