# Supplementary material for: The gut microbiome drives inter- and intra-individual differences in metabolism of bioactive small molecules
Source: Sci Rep. 2020 Nov 11;10:19590. doi: 10.1038/s41598-020-76558-5 (PMC7658971; doi:10.1038/s41598-020-76558-5)
Supplement: Supplementary file 1 — Supplementary Information. [file 41598_2020_76558_MOESM1_ESM.docx]

**Supplementary information**

The gut microbiome drives inter- and intra-individual differences in metabolism of bioactive small molecules

Asimina Kerimi^1,2^, Nicolai U. Kraut^2^, Joana Amarante da Encarnacao^2^, Gary Williamson^1,2^

^1^Department of Nutrition, Dietetics and Food, School of Clinical Sciences at Monash Health, Faculty of Medicine, Nursing and Health Sciences, Monash University, Notting Hill BASE facility, 264 Ferntree Gully Road, Notting Hill, VIC 3168, Australia

^2^School of Food Science and Nutrition, University of Leeds, Leeds, LS2 9JT, UK

Correspondence to G Williamson ([gary.williamson1@monash.edu](mailto:gary.williamson1@monash.edu))

Keywords: microbiome; coffee; personalised nutrition; phenolic; metabolism

**Supplementary Figure 1: Chemical structures of phenolic acids and conjugates**

**Supplementary Figure 2. Gender differences in biomarkers of health**.

The differences between genders for the concentration of selected biomarkers of health were tested by either an independent t-test (A to C) or a Mann-Whitney U-test (D to F). UA, uric acid.○: non-extreme outlier (the data point is > 1.5 box-length away from the corresponding plotted box edge), ●: extreme outlier (the data point is > 3 box-lengths away from the corresponding plotted box edge).

**Supplementary Figure 3: Appearance of metabolites in urine over time after coffee consumption.**

Urine was collected after coffee consumption (data shown for 2^nd^ visit, with n = 46, mean ± standard deviation). A = DHCA-3'-sulfate, B = FA-gly, C = VA-gly, D = FA-4'-sulfate, E = DHFA-4'-sulfate, F = DHFA.

**Supplementary Figure 4: Urinary excretion of phenolic acid metabolites.** Data are shown in urine for each individual (n = 36) after coffee consumption. Visit 1, black bars; visit 2, dark grey bars; visit 3, light grey bars. (a) VA-gly and FA-gly. (b) FA-4'-sulfate, DHCA-3'-sulfate, DHFA-3'-sulfate. (c) DHFA and the sum of all 6 metabolites.

**Supplementary Figure 5: Chromatographic analysis of aminothiols after derivatization.**

Panel A shows standards 1-5 run on three different days. Panel B shows the typical chromatographic profile of aminothiols in 0.53 μL of fasting plasma in a healthy adult, with inset showing an expanded y axis. The numbered peaks are: 1, Cys (500 pmol in panel A); 2, HCys (125 pmol); 3, CysGly (250 pmol); 4, GSH (125 pmol) and 5, internal standard cystamine dihydrochloride (60 pmol in panel A and B).

**Supplementary Table 1: Urinary phenolic acid metabolites as a percentage of dose**

| **Metabolite** | **This study** | **Stalmach *et al.*, 2009** **(****78)** | **Stalmach *et al.*, 2014** **(****80)** |
| --- | --- | --- | --- |
| **DHCA-3'-sulfate** | 6.8 | 9.0 | 6.0 |
| **FA-gly** | 5.9 | 5.0 | 3.4 |
| **VA-gly** | 4.8 | n.a. | n.a. |
| **FA-4'-sulfate** | 4.6 | 2.7 | 2.0 |
| **DHFA-4'-sulfate** | 3.1 | 3.0 | 3.4 |
| **DHFA** | 2.2 | 2.4 | 1.3 |
| **Sum of all metabolites** | 27.3 | 22.1 | 16.2 |

Comparison of urinary metabolites shown as a percentage of the dose ingested. In this study, urine was collected for 36 h (n = 36) after the consumption of coffee in contrast to 24 h for the other two studies. n.a. = compound not analyzed.

**Supplementary Table 2: Correlation between anthropometric data and blood biomarkers.**

|  |  | **Cys** | **HCys** | **CysGly** | **CysGly:Cys** | **GSH** | **GSH:CysGly** | **Uric acid** | **Glucose** | **Insulin** |
| --- | --- | --- | --- | --- | --- | --- | --- | --- | --- | --- |
| **Body mass index** | r_s_  *p* | *0.319  0.013 | **0.334  0.009 | ns  ns | ns  ns | *-0.278  0.031 | ns  ns | *0.274  0.034 | **0.395  0.002 | *0.266  0.040 |
| **Waist to hip ratio** | r_s_  *p* | *0.319  0.013 | *0.321  0.012 | ns  ns | ns  ns | ns  ns | ns  ns | ***0.497  <0.0005 | ns  ns | ns  ns |
| **Systolic blood pressure** | r_s_  *p* | ns  ns | **0.363  0.005 | **0.396  0.002 | *0.297  0.023 | ns  ns | *0.297  0.023 | ns  ns | ns  ns | ns  ns |

Data from the first visit (n = 60) since the parameters did not differ significantly between the 3 visits. r_s_: Spearman’s coefficient; *p* ≤ 0.05*, *p* ≤ 0.01**, *p* ≤ 0.001. ns = no significant relationship. No significant relationship between age and blood biomarkers was seen. BMI, body mass index.

**Supplementary Table 3: Chromatographic settings and multiple reaction monitoring of phenolic acid conjugates.**

| **Compound** | **Time window**  **(min)** | **Retention time (min)** | **Precursor ion**  **(m/z)** | **Fragmentor energy**  **(V)** | **Product ion**  **(m/z)** | **Collision energy**  **(V)** | **Limit of detection (pmol)** | **Limit of quantitation (pmol)** |
| --- | --- | --- | --- | --- | --- | --- | --- | --- |
| **VA-gly** | 3.0-  4.5 | 4.2 | 224 | 90 | 100  123  108 | 6  10  22 | 4 | 12 |
| **DHCA-3′-sulfate** | 4.5-  7.0 | 5.8 | 261 | 90 | 181  137  109 | 12  18  26 | 6 | 17 |
| **DHFA-4′-sulfate**  **FA-4′-sulfate** | 7.0-  15.0  7.0-  15.0 | 8.6  10.7 | 275  273 | 100  90 | 195  136  80  193  178  134 | 10  16  14  8  16  26 | 4  5 | 13  16 |
| **FA-gly** | 15.0-16.0 | 15.6 | 250 | 90 | 206  163  134 | 6  20  18 | 1 | 02 |
| **DHFA** | 16.0-16.5 | 16.2 | 195 | 90 | 136  121  93 | 12  26  30 | 5 | 15 |
| **Sinapic acid (internal standard)** | 16.5-19.5 | 16.7 | 223 | 90 | 208  193  164 | 6  16  10 | 3 | 8 |
