# Supplementary figures and images for: The gut microbiome drives inter- and intra-individual differences in metabolism of bioactive small molecules
Source: Sci Rep. 2020 Nov 11;10:19590. doi: 10.1038/s41598-020-76558-5 (PMC7658971; doi:10.1038/s41598-020-76558-5)

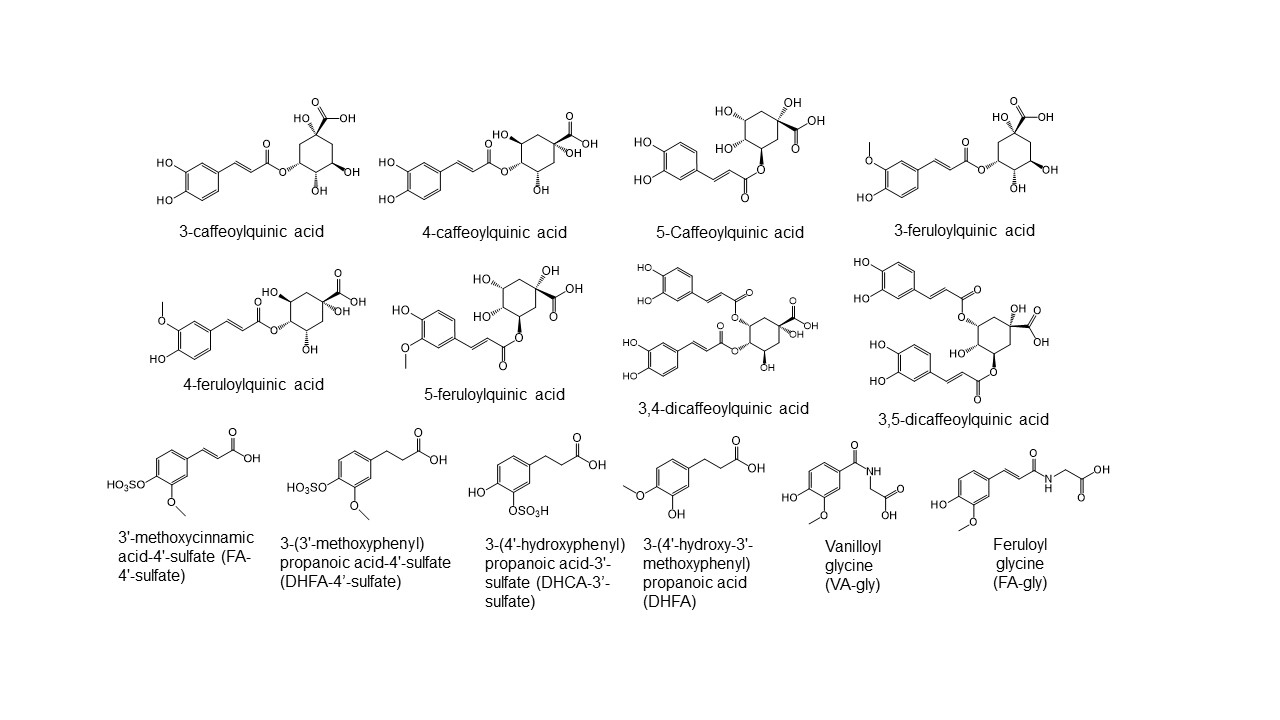

Supplement: Supplementary file 2 — Supplementary Figure s1. [file 41598_2020_76558_MOESM2_ESM.jpg]

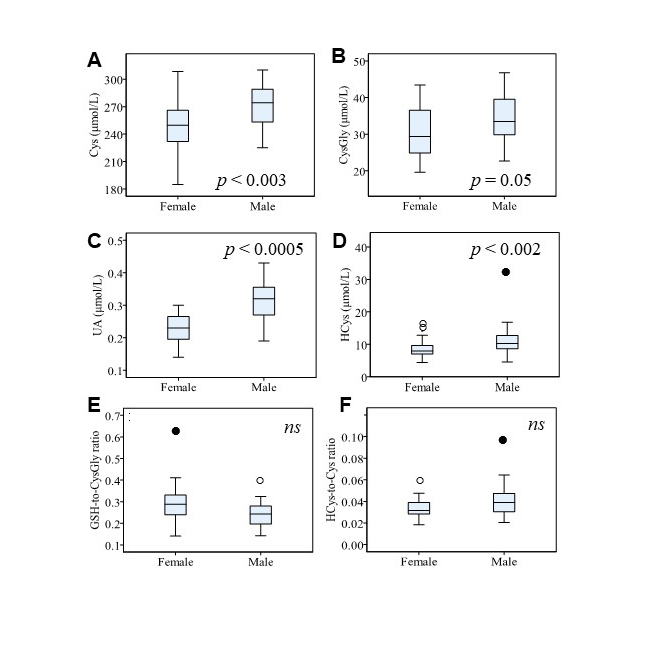

Supplement: Supplementary file 3 — Supplementary Figure s2. [file 41598_2020_76558_MOESM3_ESM.jpg]

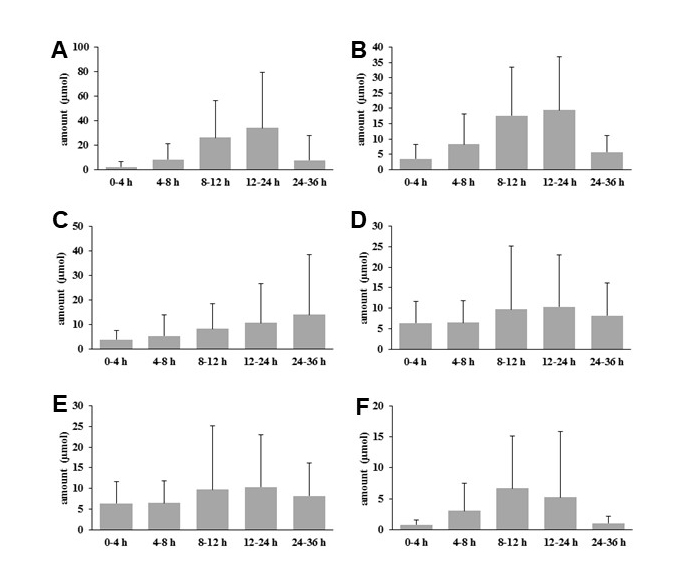

Supplement: Supplementary file 4 — Supplementary Figure s3. [file 41598_2020_76558_MOESM4_ESM.jpg]

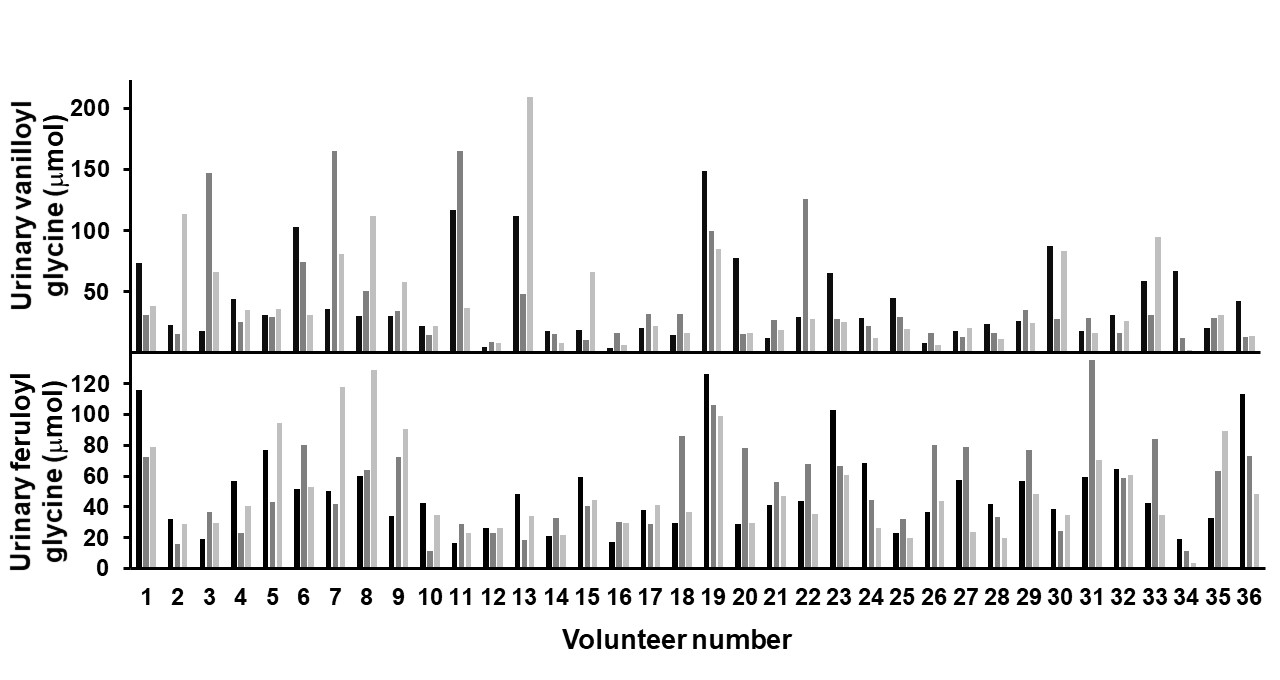

Supplement: Supplementary file 5 — Supplementary Figure s4a. [file 41598_2020_76558_MOESM5_ESM.jpg]

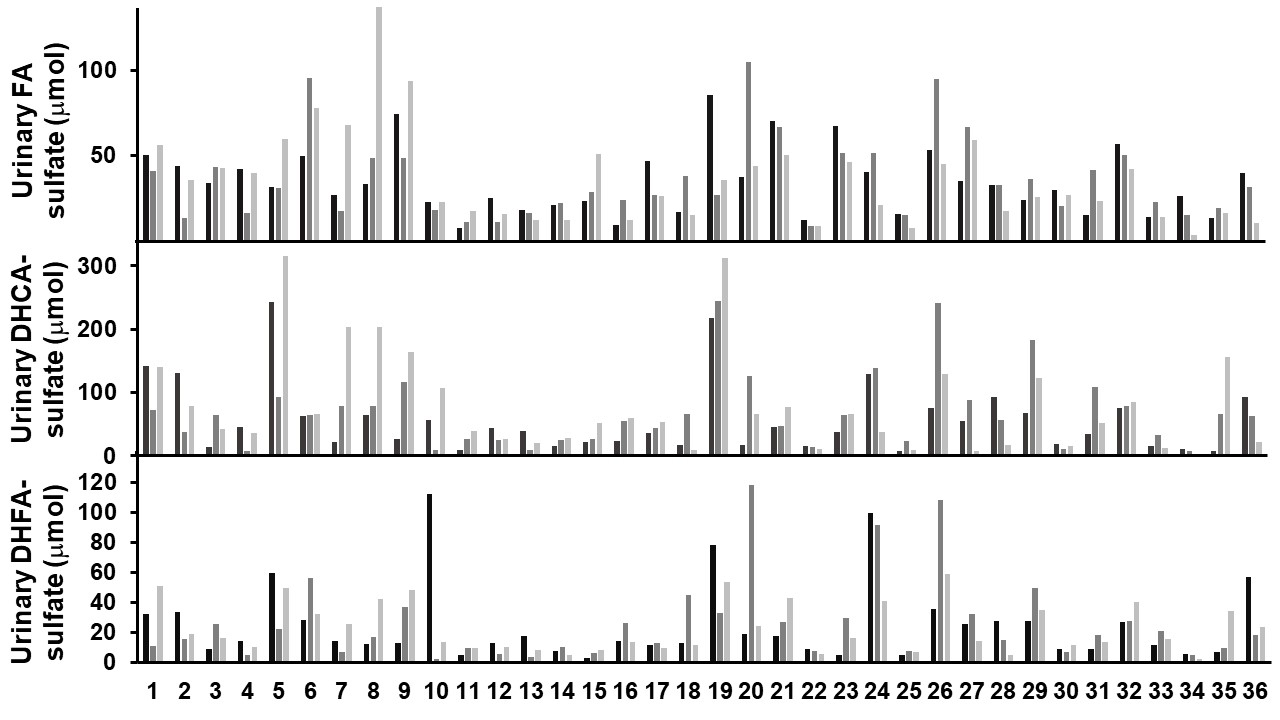

Supplement: Supplementary file 6 — Supplementary Figure s4b. [file 41598_2020_76558_MOESM6_ESM.jpg]

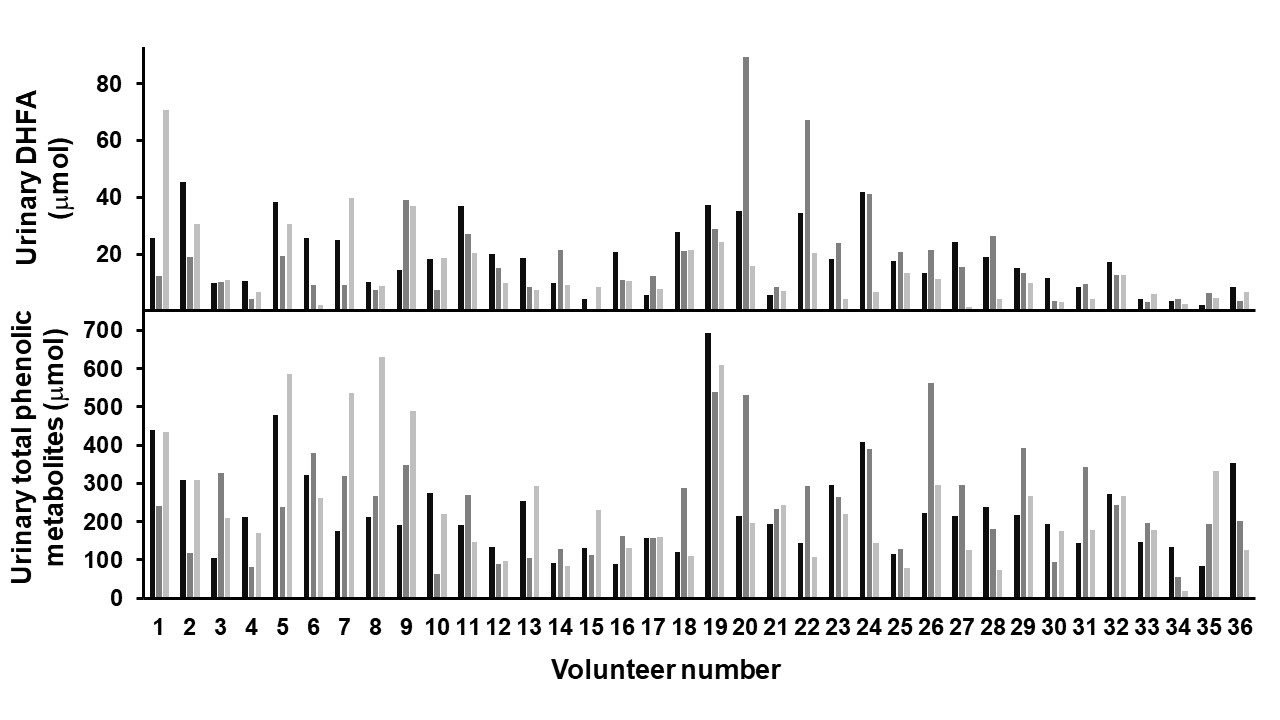

Supplement: Supplementary file 7 — Supplementary Figure s4c. [file 41598_2020_76558_MOESM7_ESM.jpg]

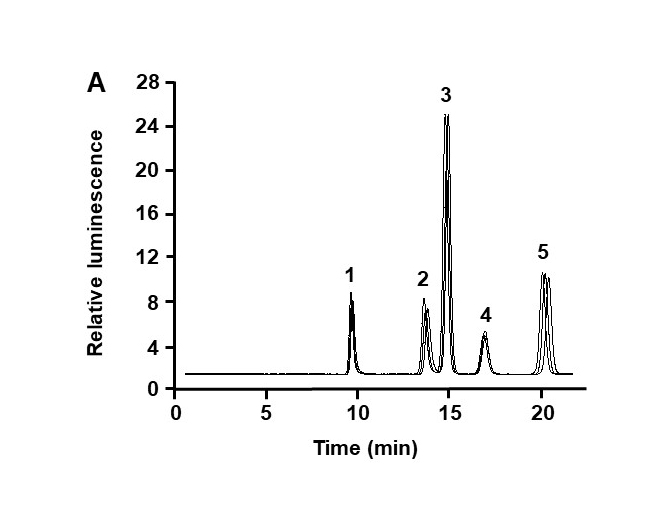

Supplement: Supplementary file 8 — Supplementary Figure s5a. [file 41598_2020_76558_MOESM8_ESM.jpg]

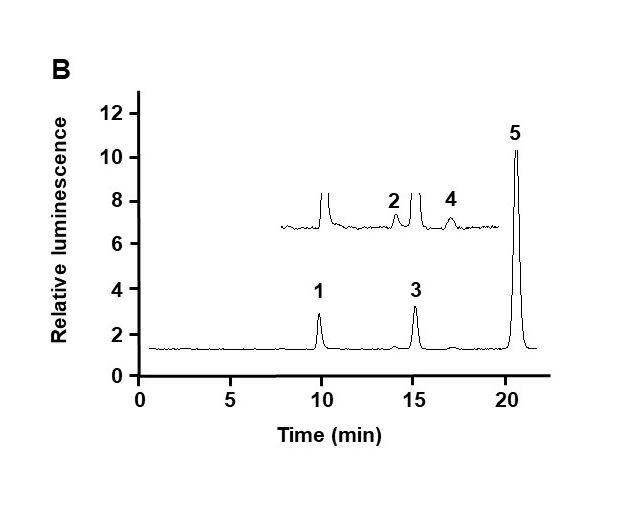

Supplement: Supplementary file 9 — Supplementary Figure s5b. [file 41598_2020_76558_MOESM9_ESM.jpg]
